# Primary hepatic neuroendocrine carcinoma coexisting with distal cholangiocarcinoma: A case report and review of the literature

**DOI:** 10.1097/MD.0000000000020854

**Published:** 2020-06-26

**Authors:** Qi Xin, Rong Lv, Cheng Lou, Zhe Ma, Gui-Qiu Liu, Qin Zhang, Hai-Bo Yu, Chuan-Shan Zhang

**Affiliations:** a Department of Pathology, Third Central Hospital of Tianjin, Tianjin Third Central Hospital affiliated to Nankai University, Tianjin Key Laboratory of Extracorporeal Life Support for Critical Diseases; b Tianjin Key Laboratory of Brain Science and Neural Engineering, Academy of Medical Engineering and Translational Medicine, Tianjin University; c Department of Radiology; d Department of Hepatobiliary Surgery, Tianjin third central hospital, Tianjin Third Central Hospital affiliated to Nankai University, Tianjin Key Laboratory of Extracorporeal Life Support for Critical Diseases; e Kidney Disease and Blood Purification Treatment Department, the Second Hospital of Tianjin Medical University, Tianjin, China.

**Keywords:** case report, cholangiocarcinoma, collision carcinoma, primary hepatic neuroendocrine carcinoma

## Abstract

**Introduction:**

Although primary hepatic neuroendocrine carcinomas, whose prognostic mechanisms remain unclear, are rare, coexistence of neuroendocrine carcinomas and other tumors is rarer. In this report, we describe a unique case of coexistence between primary hepatic neuroendocrine carcinoma and a distal cholangiocarcinoma in the pancreas.

**Patient concerns:**

A 64-year-old woman with a history of diabetes, but none of hepatitis, was admitted to hospital because of intermittent epigastric distension and pain discomfort for more than 1 month aggravated 1 day. A contrast-enhanced computed tomography (CT) scan of the upper abdomen and abdominal magnetic resonance imaging (MRI) revealed a thickening of the bile duct wall in the middle and lower segment of common bile duct and the corresponding lumen is narrow and low-density tumors with ring enhancement (1.83 cm × 1.9 cm) in lobi hepatis dexte.

**Diagnosis:**

Primary neuroendocrine carcinoma of the liver was diagnosed to be coexisting with a distal cholangiocarcinoma, which had invaded the pancreas. Immunohistochemical examination revealed that the neoplastic cells strongly expressed chromogranin A, synaptophysin, and CD56 proteins. The tumor cells did not express HepPar-1, glypican-3, S-100, CK7, and CK19 in the liver tumor. A distal bile duct in pancreatic tissues shows the characteristics of typical bile duct carcinoma, as an invasion of carcinoma is also seen in the pancreatic tissues. Gastrointestinal endoscopy, chest and abdominal CT, abdominal MRI, and positron emission tomography (PET)-CT were used to exclude metastatic neuroendocrine tumors of the liver.

**Interventions:**

Resection of the pancreas-duodenum, the right anterior lobe of the liver, and regional lymph nodes was performed in patients.

**Outcomes:**

The patient had survived for 5 months after the operation.

**Conclusion:**

A unique case of a coexistence of primary hepatic neuroendocrine carcinoma and a distal cholangiocarcinoma, which had invaded the pancreas. No treatment guidelines are established for the treatment of the unique case.

## 1 Introduction

Primary hepatic neuroendocrine neoplasms (PHNEN) are rare, accounting for 0.3% of neuroendocrine tumors and 0.28% to 0.46% of malignant liver tumors.^[1]^ Rarer is the mixed entity of neuroendocrine carcinoma and another tumor. There have been 23 reported cases of a neuroendocrine carcinoma (NEC) coexisting with a hepatocellular carcinoma (HCC) in the same patient in the literature till date.^[2–20]^ One other report shows coexisting cancers: Hepatic neuroendocrine carcinomas with gall bladder adenocarcinoma.^[10]^One case of a primary hepatic NEC combined with a cholangiocellular carcinoma (CCC) in 1 nodule has been reported.^[11]^ In this report, we describe a unique case of a coexistence of primary hepatic neuroendocrine carcinoma and distal cholangiocarcinoma in the pancreas, which had invaded the pancreas.

## 2 Case report

**Figure 1:**
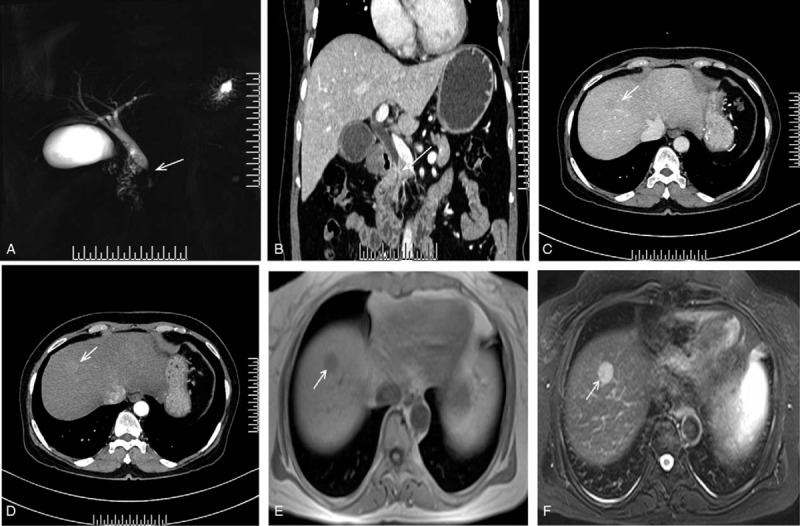
A, A MRI scan of the upper abdomen revealed the bile duct lumen is narrow (white arrow). B, A CT revealed a thickening of the bile duct wall in the middle and lower segments of common bile duct (white arrow). C, A CT scan of liver showing a low-density tumors with ring enhancement in portal phase (white arrow). D, A CT scan of liver showing a low-density tumor with ring enhancement in arterial phase (white arrow). E, Long T1 and long T2 signal nodular shadows in the parietal hepatic region with clear boundaries (white arrow). F, Long T1 and long T2 signal nodular shadows in the parietal hepatic region with clear boundaries (white arrow). CT = contrast-enhanced computed tomography, MRI = magnetic resonance imaging.

A 64-year-old woman, with a history of diabetes, tested negative for hepatitis B and C serological tests. He was admitted to hospital in April 2019 after her experience of intermittent epigastric distension and pain discomfort for more than 1 month aggravated 1 day. Results of the routine blood tests, including liver function tests, are as follows: 36 μ/L aspartate aminotransferase, 66 U/L alanine aminotransferase, 225 U/L alkaline phosphatase, 144 μ/L γ-glutamyltranspetidase, 1.93 mmol/L glycerin trilaurate, 70.57 μ/L, and carbohydrate antigen (CA) 19-9. Endoscopic retrograde cholangiopancreatography showed stenosis of the distal intrapancreatic bile duct. A computed tomography (CT) scan of the upper abdomen and magnetic resonance imaging (MRI) revealed a thickening of the bile duct wall in the middle and lower segment of common bile duct and the corresponding lumen is narrow (Fig. 1, A and B, white arrow). She was thought to have a distal cholangiocarcinoma. She also had low-density tumors with ring enhancement in lobi hepatis dexte in a CT scan (Fig. 1, C and D, white arrow), long T1, and long T2 signal nodular shadows in the parietal hepatic region with clear boundaries in a MRI (Fig. 1, E and F, white arrow). The size of the lesion was 1.827 cm × 1.911 cm. This mass in the liver was thought to be a neuroendocrine tumor. No masses were found in the gall bladder, kidney, and spleen. The patient underwent the Whipple and had been resected the right anterior lobe of the liver and regional lymph nodes.

**Figure 2:**
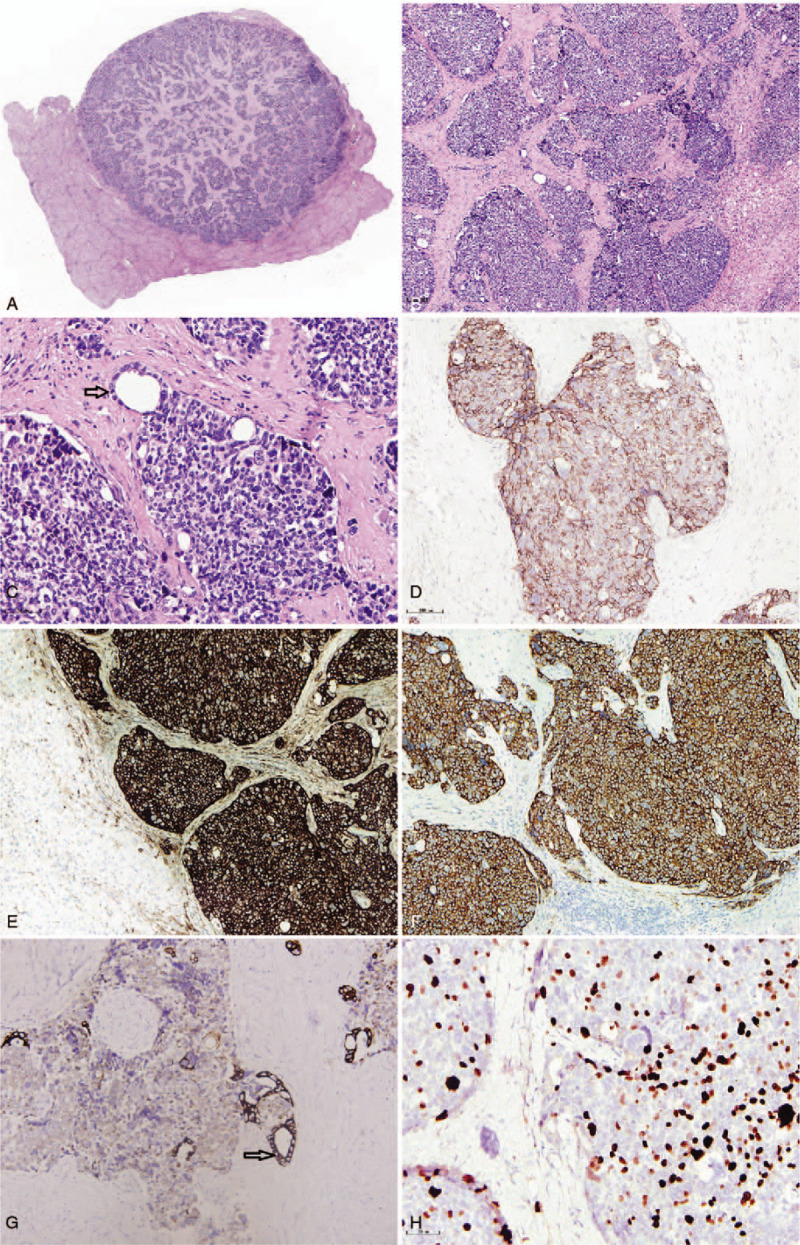
A, Well-defined nodules, consisting of nesting cells, were seen in the liver tissue. B, C, Nests or sheets of neoplastic cells with round to oval hyperchromatic nuclei with frequent mitotic figures and eosinophilic cytoplasm, residual bile ducts are visible in the margin of the cancer nest (black arrow) ×20. D–H, Immunohistochemistry, the neuroendocrine carcinoma component is focally positive for (D) CD56, (E) chromogranin, and (F) synaptophysin ×20. G, CK7 shows residual bile duct on the edge of cancer nest (black arrow) × 20. H, Ki-67 index ×40.

**Figure 3:**
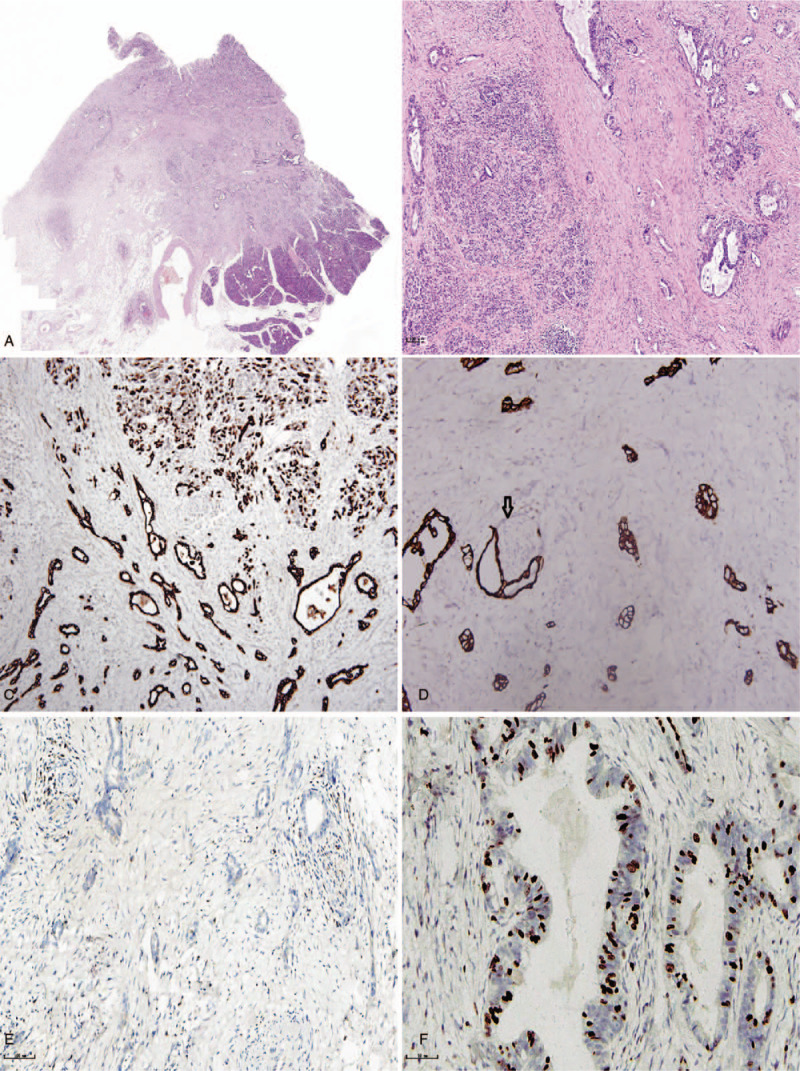
A, Tumor cells invade pancreas, duodenal mucosa under low power microscope. B, The computerized tissue of atrophied pancreas shows moderately differentiated adenocarcinoma ×20. C, Positive expression of CK7 in cancer tissues ×20. D, CK19 shows that cancer tissue invades nerve ×20. E, Negative expression of Cga in carcinoma tissue by immunohistochemical staining ×20. F, Immunohistochemistry Ki-67 index ×40.

On pathological examination, the hepatic tumor was greyish-white, hard, and well-circumscribed, with a diameter of 2 cm. The partial thickening of the bile duct wall in the pancreas was not clear about the relationship between the bile duct wall and pancreas. The length of the thickened bile duct was 3 cm. Histological evaluation of the hepatic tumors had nests or sheets of neoplastic cells with round to oval hyperchromatic nuclei, frequent mitotic figures, and eosinophilic cytoplasm; residual bile ducts were visible in the margin of the cancer nest (Fig. 2, A and B). Immunohistochemical examination revealed that the neoplastic cells strongly expressed chromogranin A, synaptophysin, and CD56 proteins (Fig. 2, C–E). The Ki-67 index was approximately 30% in the most proliferative tumor regions (Fig. 2F). The tumor cells did not express HepPar-1, glypican-3, S-100, CK7, and CK19 (data not shown). They showed characteristics of a typical bile duct carcinoma in pancreatic tissues. In the abundant fibrous stroma, an irregular tubular or adenoid structure composed of distinctly-shaped cells was found, which had a glandular tube that was irregular and branched. The core of the cancer cells disappeared, and the peripheral invasion of the nerve could be seen as in pancreatic acinar atrophy (Fig. 3A). No neuroendocrine cell proliferation was seen. Immunohistochemical examination revealed that the neoplastic cells strongly expressed CK7 and CK19, but negative for chromogranin A and synaptophysin (Fig. 3, E–G). No cancer tissue was found in the stomach, gallbladder, duodenum, and common hepatic duct. No cancer metastasis was also found in the lymph nodes (0/ 7). Gastrointestinal endoscopy, chest and abdominal CT, abdominal MRI, and positron emission tomography (PET)-CT were used to exclude metastatic tumor of the liver. Based on all the results, we made a diagnosis of primary neuroendocrine carcinoma of the liver, and the histological grading is Glade 3, with an invasion of distal cholangiocarcinoma into the pancreas. The staging of the distal cholangiocarcinoma is T_3_N_0_M_0,_ and the histologic grading revealed a moderately differentiated adenocarcinoma. The patient had since survived for 5 months after the operation.

## 3 Literature review

Twenty-six articles with well-documented coexistence primary hepatic neuroendocrine carcinoma and other tumor cases were reviewed; all articles were published between 1984 and May 2019. Table 1 describes the epidemiology, clinical presentation, surgical treatment, and outcome of these studies.^[2–4,6–23]^ According to the data reviewed, this kind of tumor is more likely to occur in middle-aged and elderly men (average age: 65.19 ± 9.65 years). Of the 27 patients described in these papers, our patient is the only female. Most cases were associated with chronic hepatitis B or C. Only 5 of the patients had no history of hepatitis, and our case had no history of hepatitis, but had a history of diabetes with mild fatty changes in liver tissues. Most cases (23/27) occur in primary hepatic neuroendocrine carcinoma and hepatocellular carcinoma concurrently, with poor prognosis. One case was uniquely associated with *EWSR1* gene rearrangement in primary hepatic neuroendocrine carcinoma and HCC.

**Table 1:**
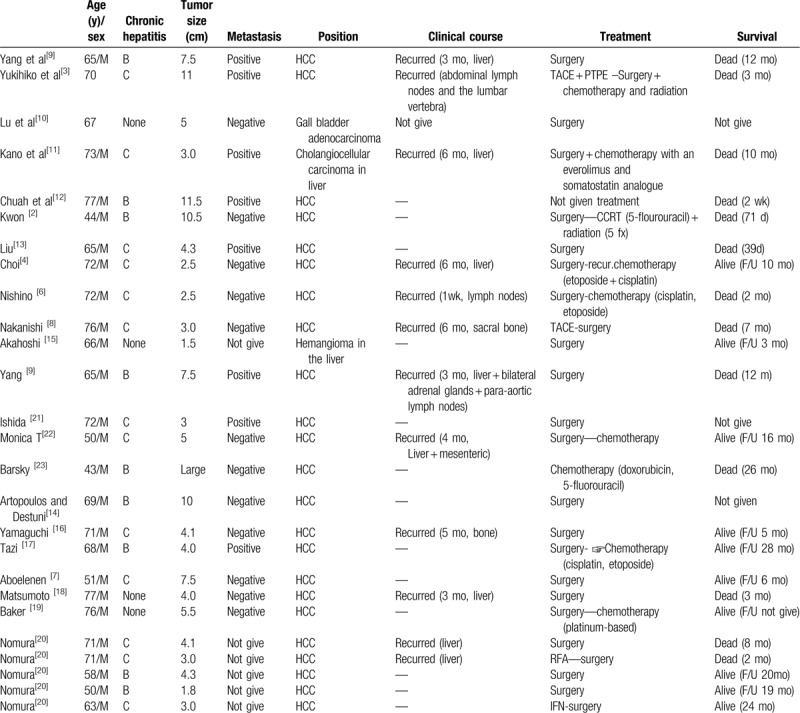
Summary of previously reported primary liver neuroendocrine mixed other carcinoma cases.

## 4 Discussion

Most neuroendocrine neoplasms occurring in the liver are metastatic lesions from other organs, such as the gastrointestinal tract and pancreas. Primary hepatic neuroendocrine neoplasms are rare. In this case, imaging and gross pathology revealed isolated masses with clear boundaries in liver tissues. In a microscopic view of a typical hepatocellular neuroendocrine carcinoma, the tumor cells are consistently small and medium-sized, cluster- or nest-shaped, arranged in pieces, deep dyeing, rope-like; they also have a small cytoplasm, a nuclear circle, a nucleus with edges and corners, and a split image. Immunohistochemical neuroendocrine markers CgA, Syn, and CD56 were strongly positive, which confirmed that the tumor originated from neuroendocrine cells, and the histological grading is Glade 3. No primary lesions were found in extrahepatic tissues, such as stomach and lungs; however, there was stenosis of the distal intrapancreatic bile duct, which was shown to be an adenocarcinoma with no neuroendocrine cancers under microscope. The gross anatomical, imaging, and histological examination confirmed the diagnosis of a primary neuroendocrine carcinoma of the liver, coexisting with a distal cholangiocarcinoma in the pancreas, which had invaded the pancreas. The staging of the distal cholangiocarcinoma is T_3_N_0_M_0,_ and the histologic grading revealed a moderately differentiated adenocarcinoma.

The origin of PHNEN can be divided into the following 3 pathways:^[24]^ neuroectodermal cells or embryonic residues scattered in the foregut or transformation of pluripotent stem cells in the original liver; ectopic pancreas or adrenal tissue; neuroendocrine cell transformation in intrahepatic bile duct epithelia. Since most PHNEN lack secretory functions similar to that of pancreatic NEN, and the study found that the bile duct contained neuroendocrine cells, one may tend to think that PHNEN originated from intrahepatic bile duct epithelia. In our case, no ectopic pancreatic tissue was found in the liver tissue, and no mass of neuroendocrine tumors was found in the resected pancreatic tissue. The CK7, CK19, and positive adenotubular structure were found in the neuroendocrine carcinoma cells of the liver; so we speculated that the primary neuroendocrine carcinoma of the liver originated from the neuroendocrine cell transformation of the intrahepatic bile duct epithelial cells.

The neuroendocrine carcinoma of the primary liver lacks specific symptoms. The diagnosis of PHNEN is based on a comprehensive analysis of symptoms, biochemistry, imaging, and histology. The diagnosis should meet 2 conditions concurrently: diagnosis by liver biopsy or surgical pathology and exclusion of primary lesions of extrahepatic tissues, such as tissues of the gastrointestinal tract, pancreas, and lungs. Immunopositivity for chromogranin A, synaptophysin, and CD56 are valuable biomarkers for pathological diagnosis and monitoring of NENs.^[25]^

No treatment guidelines are established for the treatment of coexisting hepatic neuroendocrine carcinoma and other carcinomas, due to the small number of cases studied. Surgical resection is the most effective therapy for a primary neuroendocrine tumor of the liver. The systemic therapies for unresectable NENs consist of biotherapy with somatostatin analogues, targeted therapies with mammalian target of rapamycin and tyrosine-kinase inhibitors, peptide receptor radionuclide therapy, and chemotherapy. Everolimus, an oral inhibitor of mammalian target of rapamycin, has been shown to exhibit anticancer activity in patients with advanced pancreatic neuroendocrine tumors and to significantly prolong progression-free survival;^[26]^ however, no evidence exists for the efficacy of everolimus in patients with an advanced NEC.^[27]^ The combination of irinotecan and cisplatin as postoperative adjuvant chemotherapy was feasible and possibly efficacious for resected large cell neuroendocrine carcinomas.^[28]^ For PHNENs, TACE as the liver-directed intra-arterial therapy has been used to relieve the tumor burden. Localized-ablative techniques (RFA, microwave, and laser ablation) could also be used in unresectable or recurrent liver lesions with diameters less than 5 cm.^[29]^ Besides, organ transplantation remains controversial as an ideal treatment, due to shortage of donors and high recurrence rates.^[30,31]^

Existing reports about PHNETs are mostly of small sample sizes, and the prognostic factors of PHNETs are still unclear. Overall survival of group PHNENs was significantly shorter than that of group PanNENs.^[25]^ Chen et al^[32]^ found that pathological classification of grade 3, high expression of Ki–67 positive index (PI), abnormal elevation of CA125, abnormalities of ALT and AST, anemia and lack of radical operation indicated a poor prognosis. High expression of Ki–67 PI was an independent prognostic factor for PHNETs. Additionally, the guiding effects of tumor size, AFP, CEA, CGA, and albumin on prognosis were limited.

This is a case of incidental primary neuroendocrine carcinoma of the liver coexisting with a distal cholangiocarcinoma, with occult onset and primary neuroendocrine carcinoma of the liver, with a diameter of 2 cm and ki-67 PI up to 30%. A distal cholangiocarcinoma invaded the peripheral pancreatic tissue and the nerves and blood vessels in the pancreas, and was histologically graded as moderately differentiated adenocarcinoma. In the previous literature, there was no report of the existence of these 2 malignant tumors in these 2 sites at the same time, and there was no standard procedure for treatment. We performed surgical resection on the patient, and the patient refused chemotherapy, and the patient died soon after surviving for 5 months.

## 5 Conclusion

We describe a unique case of a coexistence of primary hepatic neuroendocrine carcinoma and a distal cholangiocarcinoma, which had invaded the pancreas. No treatment guidelines are established for the treatment of the unique case.

## Author contributions

Qi Xin, Zhe Ma, Gui-Qiu Liu, and Cheng Lou collected the patients, clinical data; Qi Xin, Qin Zhang, Rong Lv, Hai-Bo Yu and Chuan-Shan Zhang designed the report and wrote the paper.

## References

[1] Song JE Kim BS Lee CH Primary hepatic neuroendocrine tumor: a case report and literature review. World J Clin Cases 2016;4:243–7. 27574614 10.12998/wjcc.v4.i8.243 PMC4983697

[2] Kwon HJ Kim JW Kim H Combined hepatocellular carcinoma and neuroendocrine carcinoma with ectopic secretion of parathyroid hormone: a case report and review of the literature. J Pathol Transl Med 2018;52:232–7. 29794961 10.4132/jptm.2018.05.17 PMC6056365

[3] Okumura Y Kohashi K Wang H Combined primary hepatic neuroendocrine carcinoma and hepatocellular carcinoma with aggressive biological behavior (adverse clinical course): a case report. Pathol Res Pract 2017;213:1322–6. 28647212 10.1016/j.prp.2017.06.001

[4] Choi GH Ann SY Lee SI Collision tumor of hepatocellular carcinoma and neuroendocrine carcinoma involving the liver: case report and review of the literature. World J Gastroenterol 2016;22:9229–34. 27895410 10.3748/wjg.v22.i41.9229 PMC5107604

[5] Yun EY Kim TH Lee SS [A case of composite hepatocellular carcinoma and neuroendocrine carcinoma in a patient with liver cirrhosis caused by chronic Hepatitis B]. Korean J Gastroenterol 2016;68:109–13. 27554219 10.4166/kjg.2016.68.2.109

[6] Nishino H Hatano E Seo S Histological features of mixed neuroendocrine carcinoma and hepatocellular carcinoma in the liver: a case report and literature review. Clin J Gastroenterol 2016;9:272–9. 27384317 10.1007/s12328-016-0669-0

[7] Aboelenen A El-Hawary AK Megahed N Right hepatectomy for combined primary neuroendocrine and hepatocellular carcinoma. A case report. Int J Surg Case Rep 2014;5:26–9. 24394859 10.1016/j.ijscr.2013.10.018 PMC3907205

[8] Nakanishi C Sato K Ito Y Combined hepatocellular carcinoma and neuroendocrine carcinoma with sarcomatous change of the liver after transarterial chemoembolization. Hepatol Res 2012;42:1141–5. 23094854 10.1111/j.1872-034X.2012.01017.x

[9] Yang CS Wen MC Jan YJ Combined primary neuroendocrine carcinoma and hepatocellular carcinoma of the liver. J Chin Med Assoc 2009;72:430–3. 19686999 10.1016/S1726-4901(09)70400-9

[10] Lu J Xiong XZ Cheng NS Hepatobiliary and pancreatic: coexisting cancers: hepatic neuroendocrine carcinomas with gall bladder adenocarcinoma. J Gastroenterol Hepatol 2014;29:1343. 25040620 10.1111/jgh.12626

[11] Kano Y Kakinuma S Goto F Primary hepatic neuroendocrine carcinoma with a cholangiocellular carcinoma component in one nodule. Clin J Gastroenterol 2014;7:449–54. 26184027 10.1007/s12328-014-0521-3

[12] Chuah KL Pang B Lim D Combined hepatocellular carcinoma and high grade neuroendocrine carcinoma with EWSR1 gene rearrangement. Pathology 2018;50:779–82. 30314647 10.1016/j.pathol.2018.07.005

[13] Liu YJ Ng KF Huang SC Composite hepatocellular carcinoma and small cell carcinoma with early nodal metastasis: a case report. Medicine 2017;96:e7868. 28834900 10.1097/MD.0000000000007868 PMC5572022

[14] Artopoulos JG Destuni C Primary mixed hepatocellular carcinoma with carcinoid characteristics. A case report. Hepatogastroenterology 1994;41:442–4. 7851852

[15] Akahoshi T Higashi H Tsuruta S Primary neuroendocrine carcinoma coexisting with hemangioma in the liver: report of a case. Surg Today 2010;40:185–9. 20107963 10.1007/s00595-009-4018-5

[16] Yamaguchi R Nakashima O Ogata T Hepatocellular carcinoma with an unusual neuroendocrine component. Pathol Int 2004;54:861–5. 15533230 10.1111/j.1440-1827.2004.01770.x

[17] Tazi EM Essadi I M’Rabti H Hepatocellular carcinoma and high grade neuroendocrine carcinoma: a case report and review of the literature. World J Oncol 2011;2:37–40. 29147223 10.4021/wjon276e PMC5649886

[18] Matsumoto H Matsukawa H Shiozaki S [A case of mixed hepatocellular and primary hepatic neuroendocrine carcinomas with remnant liver recurrence and rapid exacerbation]. Gan To Kagaku Ryoho 2017;44:1748–50. 29394763

[19] Baker E Jacobs C Martinie J Mixed Hepatocellular Carcinoma. Neuroendocrine Carcinoma of the Liver. Am Surg 2016;82:1121–5. 28206942

[20] Nomura Y Nakashima O Akiba J Clinicopathological features of neoplasms with neuroendocrine differentiation occurring in the liver. J Clin Pathol 2017;70:563–70. 27881473 10.1136/jclinpath-2016-203941

[21] Ishida M Seki K Tatsuzawa A Primary hepatic neuroendocrine carcinoma coexisting with hepatocellular carcinoma in hepatitis C liver cirrhosis: report of a case. Surg Today 2003;33:214–8. 12658390 10.1007/s005950300048

[22] Garcia MT Bejarano PA Yssa M Tumor of the liver (hepatocellular and high grade neuroendocrine carcinoma): a case report and review of the literature. Virchows Arch 2006;449:376–81. 16896889 10.1007/s00428-006-0251-0

[23] Barsky SH Linnoila I Triche TJ Hepatocellular carcinoma with carcinoid features. Hum Pathol 1984;15:892–4. 6147306 10.1016/s0046-8177(84)80152-5

[24] Gravante G Carino NDL Overton J Primary carcinoids of the liver: a review of symptoms, diagnosis and treatments. Dig Surg 2008;25:364–8. 18984960 10.1159/000167021

[25] Li MX Li QY Xiao M Survival comparison between primary hepatic neuroendocrine neoplasms and primary pancreatic neuroendocrine neoplasms and the analysis on prognosis-related factors. Hepatobiliary Pancreat Dis Int 2019;18:538–45. 30981633 10.1016/j.hbpd.2019.03.009

[26] Yao JC Pavel M Lombard-Bohas C Everolimus for the treatment of advanced pancreatic neuroendocrine tumors: overall survival and circulating biomarkers from the randomized, Phase III RADIANT-3 study. J Clin Oncol 2016;34:3906–13. 27621394 10.1200/JCO.2016.68.0702 PMC5791842

[27] Long KB Srivastava A Hirsch MS PAX8 Expression in well-differentiated pancreatic endocrine tumors: correlation with clinicopathologic features and comparison with gastrointestinal and pulmonary carcinoid tumors. Am J Surg Pathol 2010;34:723–9. 20414099 10.1097/PAS.0b013e3181da0a20

[28] Kenmotsu H Niho S Ito T A pilot study of adjuvant chemotherapy with irinotecan and cisplatin for completely resected high-grade pulmonary neuroendocrine carcinoma (large cell neuroendocrine carcinoma and small cell lung cancer). Lung Cancer 2014;84:254–8. 24679951 10.1016/j.lungcan.2014.03.007

[29] Uri I Avniel-Polak S Gross DJ Update in the therapy of advanced neuroendocrine tumors. Curr Treat Options Oncol 2017;18:72. 29143892 10.1007/s11864-017-0514-9

[30] Moris D Tsilimigras DI Ntanasis-Stathopoulos I Liver transplantation in patients with liver metastases from neuroendocrine tumors: a systematic review. Surgery 2017;162:525–36. 28624178 10.1016/j.surg.2017.05.006

[31] Clift AK Frilling A Liver transplantation and multivisceral transplantation in the management of patients with advanced neuroendocrine tumours. World J Gastroenterol 2018;24:2152–62. 29853733 10.3748/wjg.v24.i20.2152 PMC5974577

[32] Chen RW Qiu MJ Chen Y Analysis of the clinicopathological features and prognostic factors of primary hepatic neuroendocrine tumors. Oncol Lett 2018;15:8604–10. 30065788 10.3892/ol.2018.8413 PMC6064769

[33] Chan ES Alexander J Swanson PE PDX-1, CDX-2, TTF-1, and CK7: a reliable immunohistochemical panel for pancreatic neuroendocrine neoplasms. Am J Surg Pathol 2012;36:737–43. 22498824 10.1097/PAS.0b013e31824aba59

